# *Pseudomonas fluorescens* F113 Can Produce a Second Flagellar Apparatus, Which Is Important for Plant Root Colonization

**DOI:** 10.3389/fmicb.2016.01471

**Published:** 2016-09-22

**Authors:** Emma Barahona, Ana Navazo, Daniel Garrido-Sanz, Candela Muriel, Francisco Martínez-Granero, Miguel Redondo-Nieto, Marta Martín, Rafael Rivilla

**Affiliations:** Departamento de Biología, Universidad Autónoma de MadridMadrid, Spain

**Keywords:** motility, pseudomonads, FlhDC, genetic island, rhizosphere

## Abstract

The genomic sequence of *Pseudomonas fluorescens* F113 has shown the presence of a 41 kb cluster of genes that encode the production of a second flagellar apparatus. Among 2,535 pseudomonads strains with sequenced genomes, these genes are only present in the genomes of F113 and other six strains, all but one belonging to the *P. fluorescens* cluster of species, in the form of a genetic island. The genes are homologous to the flagellar genes of the soil bacterium *Azotobacter vinelandii*. Regulation of these genes is mediated by the *flhDC* master operon, instead of the typical regulation in pseudomonads, which is through *fleQ*. Under laboratory conditions, F113 does not produce this flagellum and the *flhDC* operon is not expressed. However, ectopic expression of the *flhDC* operon is enough for its production, resulting in a hypermotile strain. This flagellum is also produced under laboratory conditions by the *kinB* and *algU* mutants. Genetic analysis has shown that *kinB* strongly represses the expression of the *flhDC* operon. This operon is activated by the Vfr protein probably in a c-AMP dependent way. The strains producing this second flagellum are all hypermotile and present a tuft of polar flagella instead of the single polar flagellum produced by the wild-type strain. Phenotypic variants isolated from the rhizosphere produce this flagellum and mutation of the genes encoding it, results in a defect in competitive colonization, showing its importance for root colonization.

## Introduction

Swimming and swarming bacterial motility are flagellum-dependent. In pathogenic bacteria, motility increases the virulence capacity of many bacterial species (Schmitt et al., 1994; Akerley et al., 1995). In plant-associated pseudomonads, motility is important to colonize the rhizosphere and full motility is a requisite for biocontrol and plant growth promotion (Chin-A-Woeng et al., 2000; Kamilova et al., 2005; Barahona et al., 2011).

Structure and function of the flagellum in *Escherichia coli* and *Salmonella enterica*, have been studied for many years. Both species show peritrichous flagella and the flagellar structure is built from inside out, requiring about 50 genes, whose expression is regulated in a hierarchy of several levels (Macnab, 2003; McCarter, 2006). At the highest level of this regulatory cascade is the master regulator *flhDC* operon, whose products, FlhC and FlhD are responsible for the flagellar regulon activation. In turn, the master regulator FlhDC is controlled by a cAMP-dependent system (Soutourina et al., 1999) which involves the CyaA adenylate cyclase and the c-AMP binding protein CRP (Botsford and Harman, 1992). A similar regulatory system has been shown for *Azotobacter vinelandii*, which also shows peritrichous flagella (Leon and Espin, 2008). The chromosome of this bacterium encodes both *fleQ* (see below) and *flhDC*, but only the later operon is required for flagella synthesis (Leon and Espin, 2008).

Within the pseudomonads, *Pseudomonas aeruginosa* and *Pseudomonas fluorescens* have been used as a model to study the synthesis and regulation of the flagellar apparatus. Conversely to enterobacteria, pseudomonads only produce one or two polar flagella. In the pseudomonads, FleQ is the master regulator (Arora et al., 1997; Dasgupta et al., 2003; Capdevila et al., 2004) and expression of most flagellar genes is controlled directly or indirectly by FleQ (Arora et al., 1997; Jyot et al., 2002; Dasgupta et al., 2003).

The sequencing of the genome of *P. fluorescens* F113 has shown that this strain encodes both types of flagella (Redondo-Nieto et al., 2012). The F113 genome contains all the genes required for the synthesis of a pseudomonads’ type flagellum, but also possesses 45 genes required for the synthesis of a second flagellum. Conversely to the operons encoding flagellar genes in pseudomonads, the region encoding the second F113 flagellum contains an *flhDC* operon (Redondo-Nieto et al., 2013). The 45 ORFs involved in the synthesis of this flagellum showed high homology to flagellar genes of *A. vinelandii* and enterobacteria. The region also showed synteny with the flagellar genes of *A. vinelandii*. The *A. vinelandii* chromosome harbors flagellar genes in two clusters, I and II. Genes in cluster I are conserved in the same order in the 41 kb region in the F113 chromosome. Cluster II in *A. vinelandii* is located 416 kb downstream of cluster I. A reduced version of this region, with its central part deleted and lacking 12 ORFs is located in an inverted orientation adjacent to cluster I in the F113 chromosome. All the genes present in *A. vinelandii* clusters, but absent in the F113 genome, encode chemotaxis proteins or proteins that are not essential for flagella synthesis (Redondo-Nieto et al., 2013).

*Pseudomonas fluorescens* F113 is able to colonize the rhizosphere of a wide variety of plants (Simons et al., 1996; Naseby and Lynch, 1999; Dekkers et al., 2000; Villacieros et al., 2003, 2005) and motility is a key trait for colonization. Hypermotile mutants (Barahona et al., 2010, 2011) or hypermotile phenotypic variants isolated from the rhizosphere (Martinez-Granero et al., 2006) are able to displace the wild-type strain in competitive colonization assays. For this reason, we have identified some genes that are part of independent regulatory pathways (Navazo et al., 2009) and regulate negatively motility, such as the two-component system GacA/S, the *kinB* and *sadB* genes, and the Wsp system. The two-component system GacA/S and the cytoplasmic protein SadB, repress the motility through *fleQ* (Navazo et al., 2009; Martinez-Granero et al., 2012). Swimming motility is also inhibited by *kinB* and the Wsp system, independently of FleQ.

In *P. aeruginosa*, KinB and AlgB form a two-component system involved in alginate synthesis (Ma et al., 1997) and also participate in the regulation of virulence and motility in this bacterium (Chand et al., 2011). A *kinB* mutant in F113 is more motile than the wild-type strain (Navazo et al., 2009). In this work, we show that the presence of the cryptic second flagellar apparatus in F113, which has homology to the *A. vinelandii* DJ, explains the hypermotility phenotype of the *kinB* mutant. The second flagellum absence in both F113 and *kinB* mutant, leads to a decrease in competitive root colonization ability.

## Materials and Methods

### Microorganisms, Growth Conditions, and Plasmids

Strains and plasmids used are listed in Supplementary Table 1. *P. fluorescens* F113 and derivatives were grown in SA medium (Scher and Baker, 1982), with shaking overnight at 28°C. Purified agar (1.5%; Pronadisa, Spain) was added for solid medium. *Pseudomonas* strains were grown in LB medium for RNA and protein extraction assays. When it was necessary, antibiotics were used at the following concentrations (μg ml^-1^): rifampicin 100; kanamycin 50; gentamicin 3; spectinomycin 100; and tetracycline 70. *E. coli* strains were grown in LB medium with shaking overnight at 37°C. In this case, antibiotics were used at the following concentrations (μg ml^-1^): kanamycin 25; gentamicin 10; ampicillin 100; spectinomycin 25; chloramphenicol 30; and tetracycline 10.

### Swimming Motility Assays

Swimming was tested on SA medium with 0.3% purified agar. Bacteria were inoculated with a sterile toothpick in plates of 50 mm in diameter containing 12 ml of SA medium and incubated at 28°C. Swimming haloes were measured after 24 h. Every assay was performed three times with four replicates each time. Average swimming halo diameters (mm) and standard deviations were calculated and all dates were compared using statistic application software SPSS. Statistical significance was calculated by the Bonferroni test of variance analysis (ANOVA; *P* < 0.05).

### Simple and Double Mutant Generation: Overexpression Assays

Mutants generation was performed by directed mutagenesis. Primers were designed (Supplementary Table 2) to amplify small fragments of *flhDC*, *fliC2*, *cyaA*, and *vfr* genes [300–500 nucleotides (nts); flhDC-F/R; fliC2-F/R; cyaA-F/R; vfr-F/R]. First, these internal fragments were cloned into cloning pGEM^®^T-easy vector and then, into the suicide vector pK18*mobsacB* (Schäfer et al., 1994) or pG18*mob*2 (Kirchner and Tauch, 2003; Supplementary Table 1). The constructs were introduced into F113 or F113-derived strains by triparental mating (in LB medium at 28°C during 16 h) using helper plasmid pRK600 (Finan et al., 1986; Supplementary Table 1) and selected for homologous recombination. The same method was used in the construction of double and triple mutants. All mutants were checked by Southern blotting and PCR. Primers were designed to amplify *flhDC* (flhDC-F2/R2; Supplementary Table 2). Overexpression of *flhDC* gene was achieved by cloning them under the control of the IPTG-inducible promoter present in the pVLT31 plasmid (de Lorenzo et al., 1993).

### Transmission Electron Microscopy

*Pseudomonas fluorescens* F113 and derivatives were grown in LB medium until optical density (OD_600_) of 0.8. Formvar-coated grids were placed on the top of a drop of bacterial cells for 30 s to allow bacterial adhesion. Grids were stained for 1 min 15 s with a 1% solution of potassium phosphotungstate and washed with a drop of sterile water (thrice for 30 s). In order to visualize the preparations, the microscope JEOL JEM 1010 (100 kV) was used (Laboratorio de Microscopía de Transmisión del Servicio Interdepartamental de Investigación, UAM) and the images were taken by the BioScan de Gatan camera. The images were analyzed through DigitalMicrograph 3.1 analyze system.

### DNA Techniques

Standard methods (Sambrook et al., 1989) were used for DNA extraction, gene cloning, plasmid preparations, and agarose gel electrophoresis. Southern blots were performed with a non-radioactive detection kit, and a chemiluminescence method was used to detect hybridization signals according to the instructions of the manufacturer (Roche Boehringer Mannheim). PCR reactions were performed using the *Tth* enzyme (Biotools) under standard conditions. DNA sequencing was done by chain-termination method using DyeDeoxy terminator cycle sequencing kit protocol as described by the manufacturer (Applied Biosystems).

### RNA Extraction, cDNA Synthesis, and Gene Expression Analysis

Total RNA was extracted using Trizol^®^ according to manufacturer’s specifications (Invitrogen) from F113 wild-type and derivatives grown at 0.8 OD_600_ in LB medium. Genomic DNA was removed by RQ1 RNase-Free DNase treatment (Promega) for 30 min at 37°C. Later, RNA was purified using Trizol^®^. The concentration of RNA was spectrophotometrically determined in a Nanodrop^®^ and integrity was verified in denaturing agarose gels. RT-PCRs were carried out using Illustra Ready-To-Go^TM^ RT-PCR Beads kit from Amersham GE Healthcare. qRT-PCRs were performed in two steps: a first step of cDNA synthesis using the SuperScript^®^III First-Strand Synthesis System from Invitrogen and a second step of qPCR using the Power SYBR^®^Green PCR Master Mix from Applied Biosystems. Relative expression was estimated as 2^-ΔΔCt^ using 16S DNA as housekeeping and wild-type ΔCt values as calibrator, as described by Livak and Schmittgen (2001). Every assay was performed three times with three replicates each time.

### Protein Extraction and Western Blots

Proteins were extracted from 200 ml exponential phase grown cultures (OD_600_ = 0.8). In order to detach the flagella, the cultures were agitated by vortexing for 2 min and then centrifuged for 15 min at 7,500 rpm and 4°C. Extracellular proteins were extracted from the supernatant by precipitation for 10 min at RT with 10% (w/v) desoxycholic acid and then, 2 h at 4°C with 10% (w/v) trichloroacetic acid, followed by two washes with chilled acetone, and were finally resuspended in Laemmli buffer (Laemmli, 1970). Proteins were resolved by 12% SDS-PAGE and stained with Coomassie blue. The same electrophoretic conditions were used for Western blotting. Acrylamide gels were transferred onto nitrocellulose membranes for 1 h under standard conditions. The membranes were incubated with a 1:10,000 dilution of an anti-flagellin antiserum (de Weger et al., 1987) for 16 h at 4°C and then with a peroxidase-tagged secondary antibody (anti-rabbit immunoglobulin) for 1 h at RT. The enhanced chemiluminescence (ECL) method and Hyperfilm ECL (Amersham Biosciences) were used for development.

### Root Competitive Colonization Assays

A modification of the “root-tip competitive colonization assay” (Simons et al., 1996) was used. Alfalfa seeds (*Medicago sativa* var. Resis) were surface disinfected in 70% ethanol for 2 min and then in diluted bleach (1:5; final sodium hypochlorite concentration, 1%) for 15 min and rinsed thoroughly with sterile distilled water. Seed vernalization was performed at 4°C for 16 h and was followed by incubation in darkness at 28°C for 1 day for germination. Germinated alfalfa seeds were sown in Leonard jar gnotobiotic systems (Villacieros et al., 2003) using ca. 3.5 l of sterile perlite as the solid substrate and 8 mM KNO_3_-supplemented FP (Fahraeus, 1957) as the mineral solution (500 ml/jar; replenished every 2 days). After 2 days, alfalfa seeds were inoculated with 10^8^ cells of the appropriate strains. In competition experiments, strains were inoculated at a 1:1 ratio. In all cases 1 ml diluted culture was inoculated per plant. Plants were maintained under controlled conditions for 2 weeks: 16 h in the light at 25°C and 8 h in the dark at 18°C. Bacteria were recovered from the rhizosphere by vortexing the root tips (last centimeter of the main root) for 2 min in a tube containing FP medium and plating the appropriate dilutions on SA plates supplemented with appropriate antibiotics. Every experiment was performed three times with three replicates each time, and every replicate contained at least 20 plants.

### Bioinformatic Analysis

*Pseudomonas fluorescens* F113 genomic sequence (NC_016830.1) from position 876600 to 961608 was used as query in Blastn v2.3.0+ (Camacho et al., 2009) to compare against complete and draft *Pseudomonas* genomes in NCBI database (nt and WGS on March 30, 2016). Own designed Python scripts were used to filter BLAST results. Genome annotations were obtained from NCBI database when possible; also RAST annotation pipeline (Aziz et al., 2008) was used. Synteny of the second flagellar region and its context was assessed from annotations and represented by using self-written Perl scripts. Protein homology was checked by Blastp (Camacho et al., 2009).

Phylogenomic distances between genomes harboring the flagellar island and close relative genomes was calculated with the genome-to-genome distance calculator (GGDC) 2.1 web service http://ggdc.dsmz.de (Meier-Kolthoff et al., 2013). Nucleotide sequence from each of the 45 ORFs from the flagellar island were retrieved from genomic annotation and aligned with Clustal Omega (Sievers et al., 2011). Resulting alignments were concatenated with own designed Python scripts following the order in *P. fluorescens* F113. The concatenated sequences were used to infer the phylogeny using maximum-likelihood method, Tamura–Nei model, and 1,000 bootstrap replicates using MEGA software (v7; Kumar et al., 2016).

Scripts are provided as **Supplementary File 1**. Statistical analyses were carried out with R software (R Core Team, 2013).

## Results

### The *Pseudomonas fluorescens* F113 Genome Encodes a Second Flagellar Apparatus in a Genomic Island

**FIGURE 1:**
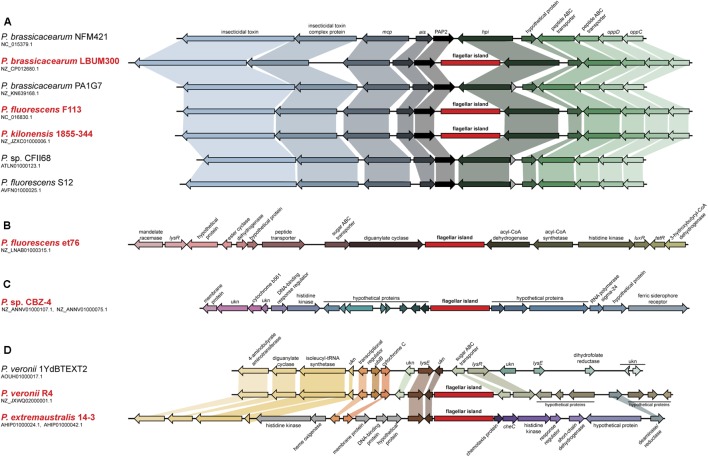
**Analysis of the second flagellar island insertion events in strains belonging to the *P. fluorescens* complex.** Syntenic context of genomes harboring the second flagellar island (red typing) and comparison with close relative genomes lacking the island (black typing). **(A)** Insertion event 1, affecting strains LBUM300, F113, and 1855-344. Close relative strains NFM421, PA1G7, CFII68, and S12, are shown for reference. **(B)** Insertion event 2, affecting strain et76. **(C)** Insertion event 3, affecting strain CBZ-4. **(D)** Insertion event 4, affecting strains R4 and 14-3. Close relative strain 1YdBTEXT2 is shown for reference. Second flagellar island is not in scale.

Analysis of the genome of *P. fluorescens* F113 showed that besides the genes required for the synthesis of the flagella, it harbors an additional 41 kb genetic region containing 45 ORFs that also showed homology with flagellar genes (Redondo-Nieto et al., 2012). This region is not present in other closely related strains belonging to the same phylogenomic group of *P. fluorescens* F113 (Redondo-Nieto et al., 2013; Garrido-Sanz et al., 2016). Syntenic comparison of the F113 genome and one of its closest sequenced relative, *Pseudomonas brassicacearum* NFM421 (**Figure 1**), showed that these 45 genes form a genetic island inserted in the intergenic region between genes PsF113_0737 and PsF113_0783 encoding a phosphatase and a peroxidase respectively, whose orthologs in *P. brassicacearum* are contiguous (PSEBRa709 and PSEBRa710). Abrupt loss of sequence homology between F113 and NFM421 indicated that the left border of the genetic island corresponds to F113 nucleotide 894337 and the right border to nucleotide 939056, the nucleotide before the stop codon of PsF113_0783. In the intergenic region between PSEBRa709 and PSEBRa710 there are 42 nts, which are present in other strains belonging to the F113 phylogenomic group but are not in the F113 genome. Those nucleotides could have been lost in F113 as result of the flagellar island insertion.

**FIGURE 2:**
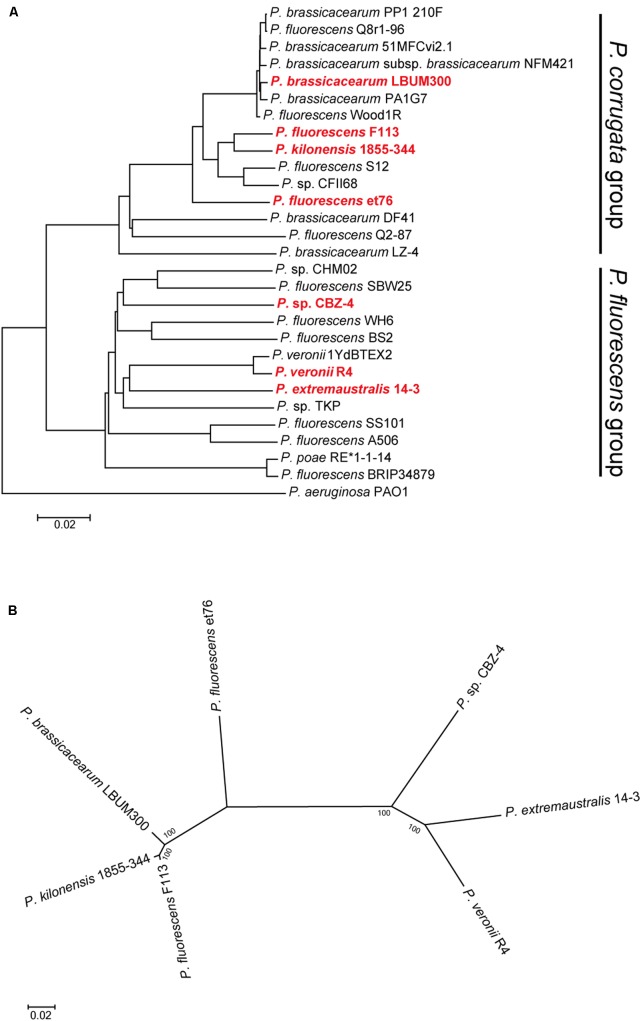
**Phylogenetic distribution of the second flagellar island in strains belonging to the *P. fluorescens* complex.** **(A)** Phylogenomic tree based on GGDC. Red bold typing indicates genomes harboring the second flagellar island. Insertion events 1 and 2, lie in strains belonging to the *P. corrugata* group, while insertion events 3 and 4, lie in strains belonging to the *P. fluorescens* group. **(B)** Co-evolution of the second flagellar island with the genomes harboring it. Multilocus sequence analysis of the 45 ORFs concatenated sequences from the second flagellar island shows agreement with the proposed four insertion events and the phylogenetic distance between the genomes harboring the island. Phylogenetic tree was built using maximum-likelihood method, Tamura–Nei model and 1,000 bootstrap replicates.

The analysis of pseudomonads genomes deposited both in the nr/nt and WGS NCBI databases, showed that besides F113, this flagellar island was also present in other seven strains, *Pseudomonas extremaustralis* 14-3b, *Pseudomonas* sp. CBZ-4, *Pseudomonas veronii* R4, *P. brassicacearum* LBUM300, *Pseudomonas kilonensis* 1855-344, *P. fluorescens* et76 and *Pseudomonas putida* ATH-43. Strains F113, 1855-344, LBUM300, and et76 are phylogenetically related and belong to the *Pseudomonas corrugata* subgroup, while strains 14-3b, CBZ-4, and R4 are also phylogenetically related and belong to the *P. fluorescens* subgroup (**Figure 2A**; Garrido-Sanz et al., 2016). Outside the *P. fluorescens* complex of species, the island was only found in a *P. putida* strain (ATH-43). The genomic location of the island is identical in strains F113, LBUM300, and 1855-344 (**Figure 1**) indicating that the presence of the island in their genomes originated from a single insertion event. The location of the island in strain et79 is in a different genomic context, indicating an independent insertion event (**Figure 1**). Similarly, the location of the flagellar island in strains R4 and 14-3 is also identical, albeit different that in the *P. corrugata* group strains (**Figure 1**). The insertion in the CBZ-4 strain is in another different region (**Figure 1**). These results indicate that within the *P. fluorescens* complex, the appearance of the flagellar island occurred in four independent insertion events. These events are also supported by a phylogenetic and phylogenomic analysis of the island (**Figure 2**). As shown in **Figure 2B** the evolution of the island is in accordance with the evolution of the genomes in which they are inserted. Furthermore, genetic organization within the island is highly conserved in all the analyzed strains (Supplementary Figure 1).

Comparison of the aminoacid sequences of the ORFs in the island present in F113 confirmed that for most of them, closest relatives outside the pseudomonads were present in *A. vinelandii* genomes. However, a few ORFs showed higher homology with ORFs present in other Gamma Proteobacteria (Supplementary Table 3).

### Genes for the Second Flagellum Are Not Expressed in *P. fluorescens* F113, but Are Expressed in a *kinB* Mutant Background

We have previously shown that ectopic expression of the *flhCD* genes in a wild-type F113 background resulted in hypermotility (Redondo-Nieto et al., 2013) indicating that the expression of the master regulatory operon is sufficient to trigger the regulatory cascade resulting in the formation of the second flagellar apparatus. However, the introduction of the plasmid was unable to restore motility to non-motile mutants such as *fliC* (data not shown), indicating that production of the first flagellum is necessary for the production of the second one or that a non-functional flagellum is produced under these conditions.

**FIGURE 3:**
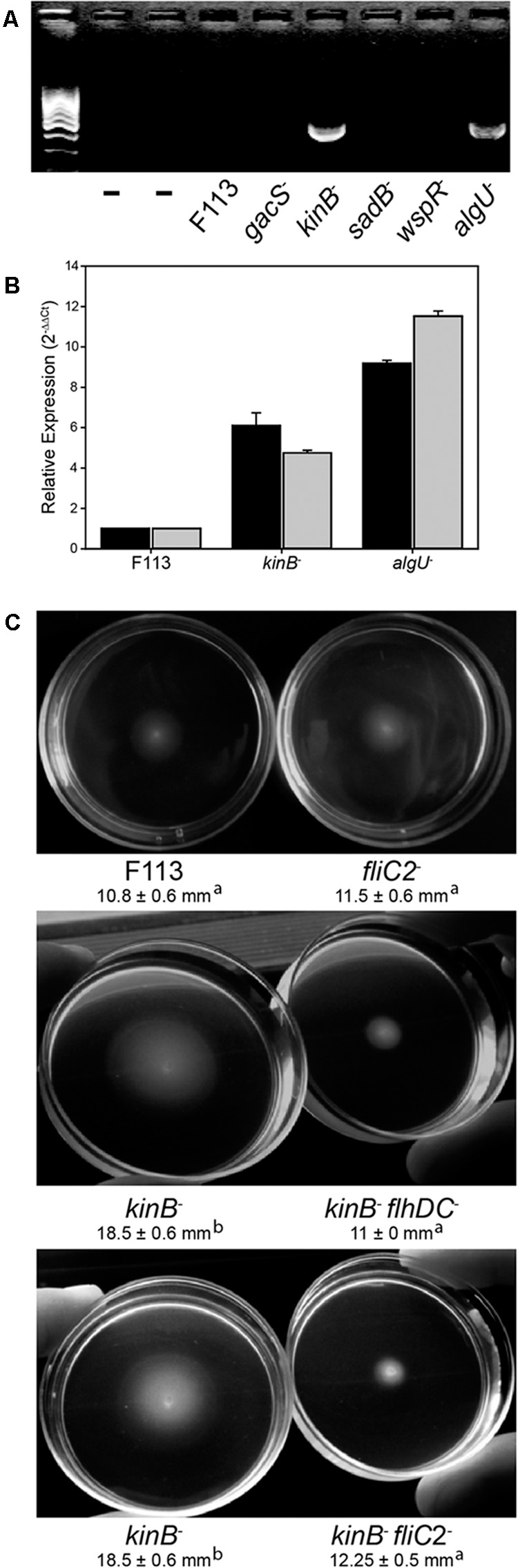
**The second flagellar apparatus is not produced in *P. fluorescens* F113, but is produced in *kinB* and *algU* mutant backgrounds.** **(A)** RT-PCR of the *fliC2* gene in *P. fluorescens* F113 and different isogenic mutants. Expression was only observed in the *kinB* and *algU* mutant backgrounds. First two lanes are negative controls (without retrotranscription) of the *kinB* and *algU* RNA preparations. **(B)** Quantitative RT-PCR analysis of the *flhDC* (black) and *fliC2* (gray) in *P. fluorescens* F113 and isogenic *kinB* and *algU* mutants. Differences between the wild-type strain and the two mutants, were statistically significant (*p* < 0.05) for both genes. **(C)** Swimming motility phenotype of *P. fluorescens* F113 and isogenic mutants affected in *fliC2*, *kinB*, *kinBflhDC*, and *kinBfliC2*, after 18 h of incubation. Different letters indicate statistically significant differences (*p* < 0.05). Motility of the strain ectopically overexpressing *flhDC* was two times (23 mm) the motility of the wild-type strain. Motility experiments were performed three times in triplicate.

In order to test the functionality of the second flagellar apparatus, we checked the expression of the *fliC2* gene encoding flagellin in F113 and isogenic mutants affected in motility (**Figure 3A**). RT-PCR experiments showed no expression of the gene in the wild-type strain, suggesting that a second flagellar apparatus is not produced by F113 under laboratory conditions. No expression was observed in the *gacS*, *sadB*, or *wspR* mutants. However, a *kinB* mutant, that had been isolated as a hypermotile mutant in a screening for motility repressors (Navazo et al., 2009) and an *algU* mutant, showed significant expression of the *fliC2* gene (**Figure 3A**). Quantitative RT-PCR was used to determine the expression of *flhDC* and *fliC2* in both mutant backgrounds compared to F113. As shown in **Figure 3B**, high level of expression was observed for both genes in both mutants. *flhDC* expression was six times higher in the *kinB* mutant and eight times higher in the *algU* mutant related to the wild-type strain. Furthermore, *fliC2* expression was 4.5 times higher in *kinB* mutant and 11 times higher in *algU* than in F113.

To test whether the hypermotility phenotype of the *kinB* mutant was due to the production of a second flagellar apparatus, we generated *flhDC* and *fliC2* mutations in a *kinB* mutant background. As shown in **Figure 3C**, both mutations suppressed the hypermotility phenotype of the *kinB* mutant, restoring wild-type motility. These results indicate that production of the second flagellar apparatus is causing the hypermotility of the *kinB* mutant. Furthermore, *fliC2* mutation in a F113 background produces no change in the motility phenotype, confirming that the second flagellum is not expressed in the wild-type strain under the conditions tested (**Figure 3C**).

### *flhDC* Are Positively Regulated by Vfr

**FIGURE 4:**
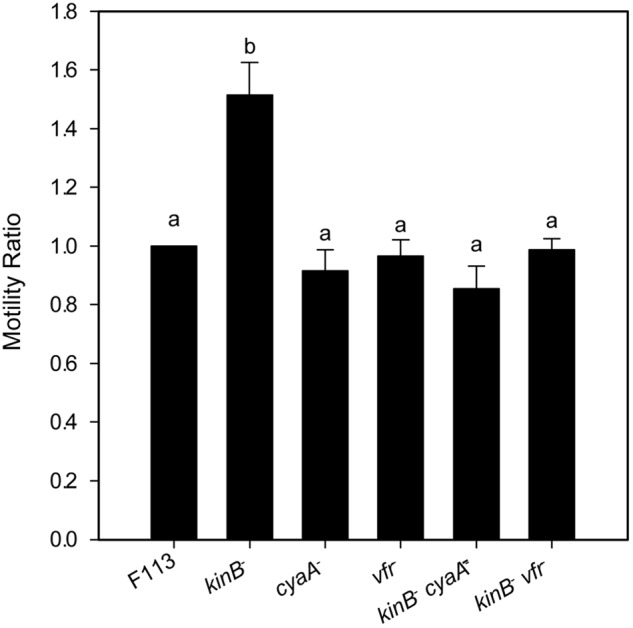
***flhDC* are positively regulated by Vfr.** Swimming motility phenotype of *Pseudomonas fluorescens* F113 and isogenic mutants affected in *kinB*, *cyaA*, *vfr*, *kinBcyaA*, and *kinBvfr*. Each strain was tested four times and means and standard deviations of measures have been represented. Only the *kinB* mutant showed significant differences (*p* < 0.05) in swimming motility compared to wild-type strain. Same letters indicate no statistical differences, *P* < 0.05.

It has been shown that in *A. vinelandii*, *flhDC* expression is activated through c-AMP by the Vfr protein (a CRP ortholog). In order to test whether this regulation was also functioning for the second flagellar apparatus in *P. fluorescens* F113, we generated *vfr* mutants in F113 and the *kinB* mutant. As shown in **Figure 4**, the *vfr* mutation had no effect in the swimming phenotype of F113. However, this mutation suppressed the hypermotile phenotype of the *kinB* mutant, restoring motility to wild-type levels. We also tested the effect of a mutation on the *cyaA* gene, encoding an adenylate cyclase, in the *kinB* background. This mutation also restored the wild-type motility phenotype to the *kinB* mutant (**Figure 4**). These results indicate that *cyaA* and *vfr* activate *flhDC* expression and therefore the production of the second flagellar apparatus and that this activation is strongly repressed by the *kinB* gene.

### Production of the Second Flagellar Apparatus Results in Polar Hyperflagellated Bacteria

**FIGURE 5:**
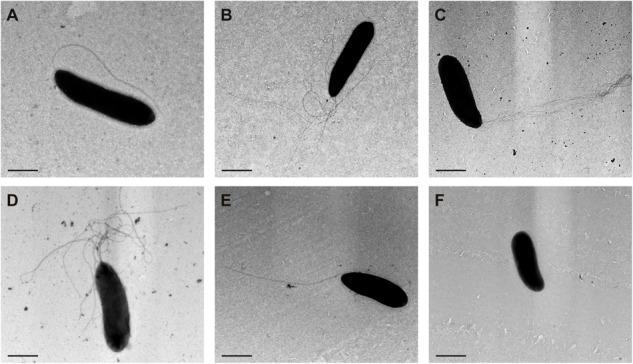
**Production of the second flagellar apparatus results in polar hyperflagellated bacteria.** Transmission electron microscopy image of **(A)** *P. fluorescens* F113; **(B)** F113 harboring a plasmid expressing *flhDC*; **(C)** *kinB* mutant; **(D)** phenotypic variant S; **(E)** *kinBfliC2* mutant; **(F)** *fliC* mutant. All the strains producing the second flagellar apparatus showed a polar tuft of flagella.

Transmission electron microscopy of negatively stained cells was used to monitor the flagellar number and location in different derivatives of *P. fluorescens* F113 (**Figure 5**). The wild-type strain (**Figure 5A**) presents a single polar flagellum, as has been previously described. Conversely, all the strains producing the second flagellar apparatus, such as the *kinB* mutant (**Figure 5C**) and the F113 (p*flhDC*; **Figure 5B**) presented a tuft of flagella in one of the poles of the cell. The same was true for the phenotypic variant S (Sánchez-Contreras et al., 2002; **Figure 5D**), a hypermotile variant isolated from the rhizosphere. A *kinB* mutant, harboring a mutation in the *fliC2* gene (**Figure 5E**) showed the same flagellation that the wild-type strain.

In order to confirm that hyperflagellation resulted from production of the second flagellar apparatus and not by hyperproduction of the first flagellum, we extracted extracellular proteins from cultures of the wild-type strain, the *kinB* mutant and a *sadB* mutant, that has been previously shown to overproduce the FliC flagellin. As shown in Supplementary Figure 2, an anti-FliC antiserum that does not recognize FliC2, showed FliC overproduction only for the *sadB* mutant.

### Production of the Second Flagellar Apparatus Is Important for Root Colonization

**FIGURE 6:**
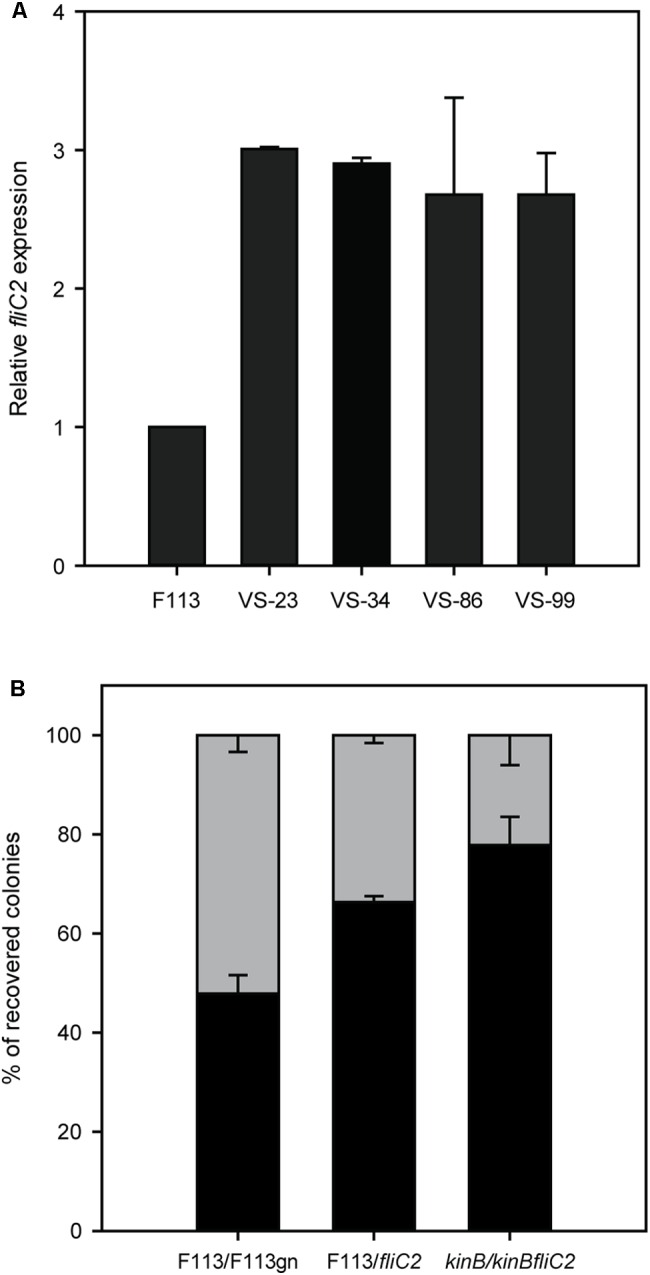
***Production* of the second flagellar apparatus is important for root colonization.** **(A)** Quantitative RT-PCR analysis of the expression of *fliC2* in *Pseudomonas fluorescens* F113 and its phenotypic variants isolated from the alfalfa rhizosphere. All the variants showed statistically significant (*p* < 0.05) differences with the wild-type strain. **(B)** Competitive root colonization assays of *Pseudomonas fluorescens* F113 and derivatives. Bacterial pairs were inoculated at a 1:1 proportion and colonies were recovered after 2 weeks of inoculation, in culture medium plates supplemented with the appropriate antibiotics. Every experiment was performed three times with three replicates each time, and every replicate contained at least 20 plants. Means and standard deviations are represented. Gray bars represent the percentage of colonies recovered from the tested strain (F113gn, *fliC2* mutant and *kinBfliC2* double mutant). Black bars represent the percentage of colonies recovered from the competitor (wild-type strains on first and second case, and *kinB* mutant on third case).

Since the phenotypic variant S appeared to be producing the second flagellar apparatus, we decided to test *fliC2* expression in a battery of hypermotile phenotypic variants isolated from alfalfa rhizosphere (Martinez-Granero et al., 2006). All the phenotypic variants expressed the *fliC2* gene (**Figure 6A**), suggesting that the rhizosphere environment selects for the production of the second flagellar apparatus. It is important to note that phenotypic variants arise at a much lower frequency from liquid medium cultures than in the rhizosphere (Martínez-Granero et al., 2005). We also tested the competitive colonization ability of the wild-type strain against a *fliC2* mutant, unable to produce the second flagella. As shown in **Figure 6B**, the *fliC2* mutant was displaced by the wild-type strain from the root tip. Furthermore, we analyzed the competitive colonization capacity of the *kinB* mutant that synthesizes both flagellum, and a *kinBfliC2* double mutant, which can only produce the first flagellar apparatus. As shown in **Figure 6B**, the *kinB* mutant was able to displace the *kinBfliC2* double mutant. Taken together, these results show that the production of the second flagella in the rhizosphere environment is an important trait for root competitive colonization.

## Discussion

Production of a second flagellar apparatus is an unusual trait in fluorescent pseudomonads. A genomic analysis has shown that within the genus *Pseudomonas* only a few strains, most belonging to the *P. fluorescens* complex of species encode the genes for its synthesis. Out of 212 sequenced strains within the complex, only seven strains contained this genetic region. Only one other sequenced pseudomonad, outside the *P. fluorescens* complex contained the genes for its synthesis. The genes encoding the synthesis of the second flagellar apparatus form a genetic island, since they are located in different genomic positions in the genomes of different strains. In the case of F113, the availability of a reference genome of a closely relative that does not contain the island, *P. brassicacearum* NFM421, has allowed to determine the precise location and boundaries of the genetic island.

Phylogenomic analysis, using whole-genome distance calculations (Meier-Kolthoff et al., 2013) showed that the seven strains belong to two of the eight subgroups that form the *P. fluorescens* complex of species (Garrido-Sanz et al., 2016): *P. corrugata* and *P. fluorescens* subgroups. The seven strains also had different origins, ranging from the plant-rhizosphere to Antarctic soil and water samples. Phylogenomic and phylogenetic analysis has shown that there have been at least five insertion events in the evolutionary story of this genetic island. It is likely that sequencing the genomes of new strains will result in finding new strains containing the flagellar island either at one of the identified locations or at a different one, representing novel insertion events. In this sense, we have recently isolated a strain RMP9, which is closely related to F113 and has the island inserted in the same position. Outside from the pseudomonads, closer homologs of the genes encoded by the genomic islands are in the *A. vinelandii* genome, although a few of them have closer relatives among different Gamma Proteobacteria. Therefore, the origin of the island is unclear.

*Pseudomonas fluorescens* F113 does not produce the second flagellar apparatus under laboratory conditions. However, the master regulator of flagella, *flhDC*, and the flagellin encoding gene, *fliC2*, are expressed in *kinB* and *algU* mutant backgrounds, indicating that both genes are strongly repressing the expression of the second flagellar genes. It has been shown that in *A. vinelandii*, motility is controlled by AlgU, which represses the *flhDC* operon (Leon and Espin, 2008). In pseudomonads, *kinB* expression requires the AlgU sigma factor (Damron et al., 2009). The results presented here in *P. fluorescens* F113 are in agreement with this data and indicate that in F113 the *flhDC* operon is under strict repression by both genes. Furthermore, we have previously shown that AmrZ, a transcriptional regulator which also requires AlgU for its transcription (Tart et al., 2005), downregulates the expression of the *flhDC* master operon (Martinez-Granero et al., 2014). Since in F113, AlgU also regulates *fleQ* transcription through repression by AmrZ (Martinez-Granero et al., 2012) it is clear that AlgU simultaneously regulates the synthesis of both flagellar apparatus in *P. fluorescens* F113, which is congruent with the proposed role of AlgU as a master regulatory gene for environmental adaption (Martinez-Granero et al., 2014).

Ectopic expression of the *flhDC* operon in F113 is enough for production of the second flagellum. When *flhDC* is expressed from a vector with a *kinB* and *algU* independent promoter, there is an increase in swimming motility, indicating the production of the second flagellar apparatus. Surprisingly, ectopic expression of *flhDC* in isogenic backgrounds unable to produce the first flagellum, did not restore motility and were aflagelated.

**FIGURE 7:**
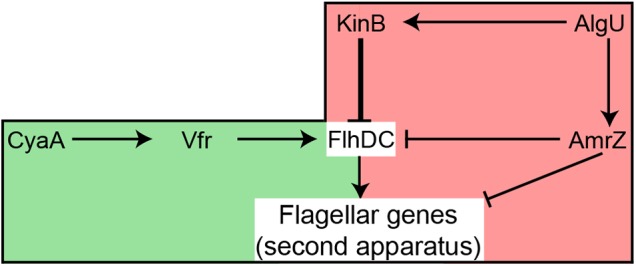
**Hypothetical model showing the regulation of the production of the second flagellar apparatus in *P. fluorescens* F113.** The flagellar master operon *flhDC* is positively regulated by Vfr, likely in response to c-AMP produced by CyaA. The master operon is strongly repressed (thick line) by KinB. AmrZ downregulates the expression of both the master operon and the flagellar structural genes. AlgU is required for the expression of *kinB* and *amrZ*. Green background indicates positive regulation, while red background indicates negative regulation of *flhDC*. Note that although AlgU and AmrZ regulate genes required for the synthesis of both flagellar apparatus, FlhDC only regulate the genes required for the second flagellar system.

An F113 *kinB* mutant is hypermotile (Barahona et al., 2011). Here we show that wild-type motility is restored by mutating either *fliC2* or *flhDC* in a *kinB*^-^ background. These results justify the phenotype of the *kinB* mutant, showing that its hypermotility is due to the production of the second flagellar apparatus, which is repressed in the wild-type background. In enterobacteria, the *flhDC* expression is under c-AMP control through the CRP protein (Stella et al., 2008). Here we have shown a similar regulatory mechanism for the biosynthesis of the second flagellar apparatus: mutation of the *vfr* gene, encoding a CRP ortholog, in a *kinB*^-^ background, resulted in suppression of the hypermotility phenotype, consistent with lack of second flagellar apparatus production. Identical result was obtained when mutating *cyaA*, which encodes an adenylate cyclase in the F113 genome. These results indicate that production of the second flagellar apparatus is dependent on c-AMP through the Vfr protein. Although in other pseudomonads it has been shown that Vfr might regulate the expression of *fleQ* (Dasgupta et al., 2002) and therefore the production of the canonical flagellar apparatus, this is not the case in F113. **Figure 7** shows a model for the regulation of the biosynthesis of the second flagellar apparatus in *P. fluorescens* F113. According to this model, the master regulatory operon for the synthesis of this flagellum, *flhDC*, is subjected to positive and negative regulation. Positive regulation is exerted through CyaA and Vfr. The *flhDC* operon is strongly repressed by KinB and downregulated by AmrZ, which also downregulates the expression of the flagellar structural genes for the synthesis of the second flagellar apparatus (Martinez-Granero et al., 2014). Both *kinB* and *amrZ* require the AlgU sigma factor for expression. Production of the second flagellar system would therefore require the relief of the KinB repression and the activation of the *flhDC* operon by Vfr and c-AMP. It should be noted that both AlgU and AmrZ are implicated in the regulation of both flagellar apparatus, indicating that these sigma factor and transcriptional regulator are key nodes in the regulation of motility in F113.

We have also shown that the second flagellar apparatus is produced as a polar tuft of flagella. This tuft was observed in the wild-type strain, which otherwise produced a single polar flagellum, when ectopically expressing the *flhDC* operon. The tuft was also observed in the *kinB* mutant. Mutation of *fliC2* in the *kinB*^-^ background restored the formation of a single polar flagellum, showing that the tuft represents the second flagellar apparatus. Furthermore, by using an anti FliC1 antiserum, we have shown that the *kinB* mutant does not produce more FliC1 than the wild-type strain. Overproduction of FliC1 was, however, observed in a *sadB* mutant, that we have previously shown that overproduces this flagellin (Navazo et al., 2009). Flagella regulated by the *flhDC* master operon are usually peritrichous, as is the case for enterobacteria and *A. vinelandii* (Leon and Espin, 2008), however, a tuft of polar flagella controlled through *flhDC* is the normal flagellation of *Burkholderia glumae* (Jang et al., 2014), indicating that flagellar location does not depend on the master regulatory genes.

The tuft of flagella was also observed in a phenotypic variant isolated from the alfalfa rhizosphere (Sánchez-Contreras et al., 2002). This observation prompted us to determine the production of the second flagellar apparatus in this environment, observing that a number of previously isolated hypermotile phenotypic variants also expressed the *fliC2* gene. These results suggested that the second flagellar apparatus might play a role in root colonization. Others and we, have previously shown that motility is one of the most important traits for competitive colonization of the rhizosphere (Barahona et al., 2011), since non-motile, non-chemotactic or mutants with reduced motility are displaced from the rhizosphere (de Weert et al., 2002; Capdevila et al., 2004). We have also observed that the rhizosphere selects for hypermotile phenotypic variants (Martinez-Granero et al., 2006). Therefore, it is likely that the hypermotility conferred by production of the second flagellar apparatus could be an important trait for competitive colonization of the root. This hypothesis was confirmed for strain F113, since blocking the production of the second flagellar apparatus by mutating the *fliC2* gene, reduced the competitive colonization ability both in a wild-type and in a *kinB* mutant background. The fact that several of the strains shown to encode the second flagellar apparatus do not come from plant environments, make it difficult to assess the ecological importance of this system in the plant-root environment. Since colonization depends in multiple traits and is highly variable even in closely related strains, experiments comparing a number of rhizosphere isolates containing the island with isogenic or nearly isogenic strains that do not contain it would be necessary to generalize the ecological importance of this flagellar system.

## Conclusion

*Pseudomonas fluorescens* F113 can produce two flagellar systems, the canonical, regulated by FleQ and a second system regulated by the *flhDC* master operon, similarly to enterobacteria and *A. vinelandii*. Genes for the synthesis of the second flagellar system are carried by a genetic island found only in a limited set of strains within the genus *Pseudomonas*. The second flagella is cryptic, but is produced by mutants in the *kinB* gene or by the wild-type strain when ectopically expressing the *flhDC* operon. Both flagella are coordinately regulated through AlgU, KinB, Vfr, and AmrZ. Production of the second flagellar apparatus in F113 results in a tuft of polar flagella, which increases motility, and gives a competitive advantage for root colonization.

## Author Contributions

EB, AN, FM-G, and CM performed experiments and analyzed results. DG-S performed bioinformatic analysis. MR-N, MM, and RR conceived and supervised the study and wrote the manuscript.

## Conflict of Interest Statement

The authors declare that the research was conducted in the absence of any commercial or financial relationships that could be construed as a potential conflict of interest.

## Supplementary Material

**SUPPLEMENTARY FILE 1 | Scripts used in the bioinformatics analysis.**
