# Supplementary material for: Pseudomonas fluorescens F113 Can Produce a Second Flagellar Apparatus, Which Is Important for Plant Root Colonization
Source: Front Microbiol. 2016 Sep 22;7:1471. doi: 10.3389/fmicb.2016.01471 (PMC5031763; doi:10.3389/fmicb.2016.01471)
Supplement: Supplementary file 1 [file Table_1.PDF]

SupplementaryTable 1. Strains and plamids used

	Charactheristics	Reference
**Strains**		
DH5α	*E. coli* cloning strain	Gibco-BRL
F113rif	*P. fluorescens* wild-type Rif^R	(Shanahan et al., 1992)
F113*sadB*-1	F113rif *sadB*$^-$mutant Rif^R Km^R	(Navazo et al., 2009)
F113*kinB*	F113rif *kinB*$^-$ mutant Rif^R Km^R	(Barahona et al., 2011)
F113*fleQ*	F113rif *fleQ*$^-$ mutant Rif^R Km^R	(Capdevila et al., 2004)
F113*fliC*	F113rif *fliC*$^-$ mutant Rif^R Km^R	(Capdevila et al., 2004)
F113*flgM*	F113rif *flgM*$^-$ mutant Rif^R Km^R	This work
F113*algU*	F113rif *algU*$^-$ mutant Rif^R Km^R	(Martinez-Granero et al., 2012)
F113*flhDC*	F113rif *flhDC*$^-$ mutant Rif^R Gn^R	This work
F113*fliC2*	F113rif *fliC2*$^-$ mutant Rif^R Gn^R	This work
F113*cyaA*	F113rif *cyaA*$^-$ mutant Rif^R Gn^R	This work
F113*vfr*	F113rif *vfr*$^-$ mutant Rif^R Gn^R	This work
F113*kinB-flhDC*	F113rif *kinB*$^-$ and *flhDC*$^-$ mutant Rif^R Km^R Gn^R	This work
F113*kinB-fliC2*	F113rif *kinB*$^-$ and *fliC2*$^-$ mutant Rif^R Km^R Gn^R	This work
F113*kinB-cyaA*	F113rif *kinB*$^-$ and *cyaA*$^-$ mutant Rif^R Km^R Gn^R	This work
F113*kinB-vfr*	F113rif *kinB*$^-$ and *vfr*$^-$ mutant Rif^R Km^R Gn^R	This work
F113*kinB-fleQ*	F113rif *kinB*$^-$ and *fleQ*$^-$ mutant Rif^R Km^R Gn^R	This work
F113*kinB-flgM*	F113rif *kinB*$^-$ and *flgM*$^-$ mutant Rif^R Km^R Gn^R	This work
F113(p*flhDC*)	F113rif containing pBG1800 Tc^R. *flhDC* overexpression	This work
F113*fliC*(p*flhDC*)	F113rif *fliC2*$^-$ mutant containing pBG1800 Tc^R Gn^R. *flhDC* overexpression	This work
F113*fleQ*(p*flhDC*)	F113rif *fleQ*$^-$ mutant containing pBG1800 Tc^R Km^R. *flhDC* overexpression	This work
Phenotypic variant S	Hypermotile phenotypic variant	(Sanchez-Contreras et al., 2002)
Phenotypic variants VS-23, VS-34, VS-86 and VS-99	Hypermotile phenotypic variants isolates of rhizosphere after Sss overexpression	(Martinez-Granero et al., 2006)
**Plasmids**		
pGEM®T-easy vector	Cloning vector Amp^R	Promega
pK18*mobsacB*	pUC18 derivative *lacZ mob* site *sacB* Km^R	(Schäfer et al., 1994)
pG18*mob*2	Suicide vector Gn^R	(Kirchner and Tauch, 2003)
pRK600	Helper plasmid Cm^R	(Finan et al., 1986)
pVLT31	IPTG-inducible expression vector Tc^R	(de Lorenzo et al., 1993)
pBG1800	pVLT31 derivative containing the *flhDC* gene (p*flhDC*) Tc^R	This work

References

Barahona, E., Navazo, A., Martinez-Granero, F., Zea-Bonilla, T., Perez-Jimenez, R.M., Martin, M., and Rivilla, R. (2011). Pseudomonas fluorescens F113 mutant with enhanced competitive colonization ability and improved biocontrol activity against fungal root pathogens. *Appl Environ Microbiol* 77**,** 5412-5419.

Capdevila, S., Martinez-Granero, F.M., Sanchez-Contreras, M., Rivilla, R., and Martin, M. (2004). Analysis of Pseudomonas fluorescens F113 genes implicated in flagellar filament synthesis and their role in competitive root colonization. *Microbiology* 150**,** 3889-3897.

De Lorenzo, V., Eltis, L., Kessler, B., and Timmis, K.N. (1993). Analysis of Pseudomonas gene products using lacIq/Ptrp-lac plasmids and transposons that confer conditional phenotypes. *Gene* 123**,** 17-24.

Finan, T.M., Kunkel, B., De Vos, G.F., and Signer, E.R. (1986). Second symbiotic megaplasmid in Rhizobium meliloti carrying exopolysaccharide and thiamine synthesis genes. *J Bacteriol* 167**,** 66-72.

Kirchner, O., and Tauch, A. (2003). Tools for genetic engineering in the amino acid-producing bacterium Corynebacterium glutamicum. *J Biotechnol* 104**,** 287-299.

Martinez-Granero, F., Navazo, A., Barahona, E., Redondo-Nieto, M., Rivilla, R., and Martin, M. (2012). The Gac-Rsm and SadB signal transduction pathways converge on AlgU to downregulate motility in Pseudomonas fluorescens. *PLoS One* 7**,** e31765.

Martinez-Granero, F., Rivilla, R., and Martin, M. (2006). Rhizosphere selection of highly motile phenotypic variants of Pseudomonas fluorescens with enhanced competitive colonization ability. *Appl Environ Microbiol* 72**,** 3429-3434.

Navazo, A., Barahona, E., Redondo-Nieto, M., Martinez-Granero, F., Rivilla, R., and Martin, M. (2009). Three independent signalling pathways repress motility in Pseudomonas fluorescens F113. *Microb Biotechnol* 2**,** 489-498.

Sanchez-Contreras, M., Martin, M., Villacieros, M., O'gara, F., Bonilla, I., and Rivilla, R. (2002). Phenotypic selection and phase variation occur during alfalfa root colonization by Pseudomonas fluorescens F113. *J Bacteriol* 184**,** 1587-1596.

Schäfer, A., Tauch, A., Jäger, W., Kalinowski, J., Thierbach, G., and Pühler, A. (1994). Small mobilizable multi-purpose cloning vectors derived from the Escherichia coli plasmids pK18 and pK19: selection of defined deletions in the chromosome of Corynebacterium glutamicum. *Gene* 145**,** 69-73.

Shanahan, P., O'sullivan D, J., Simpson, P., Glennon, J.D., and O'gara, F. (1992). Isolation of 2,4-diacetylphloroglucinol from a fluorescent pseudomonad and investigation of physiological parameters influencing its production. *Appl Environ Microbiol* 58**,** 353-358.
