# Supplementary material for: Pseudomonas fluorescens F113 Can Produce a Second Flagellar Apparatus, Which Is Important for Plant Root Colonization
Source: Front Microbiol. 2016 Sep 22;7:1471. doi: 10.3389/fmicb.2016.01471 (PMC5031763; doi:10.3389/fmicb.2016.01471)
Supplement: Supplementary file 2 [file Table_2.PDF]

Supplementary Table 2. Primers used

Primer	Sequence (5´-3´)	Annealing temperature (°C)	Reference
cyaA-F	GCTCTGGCAACTGTTCAAAGG	58	This work
cyaA-R	CTGTCCAGCAGCGCCAGTT	58	This work
flhDC-F	AAAAGCCTGGTCACCGAAATG	58	This work
flhDC-R	CTTGATGACCGACCTGTTCCA	58	This work
flhDC-F2	CCCAGTAAGGGAAAAGCGAAA	58	This work
flhDC-R2	ACGCCTTGTGGTAAAGGGGTA	58	This work
fliC2-F	CGTCCGGTCTGAAAATCAACA	58	This work
fliC2-R	CGTAGCTGGTGTTGGTCGAAG	58	This work
vfr-F	TTCCGACACGTCGTTTTTCAT	58	This work
vfr-R	ATCGGGTTGCTTGCACAGTT	58	This work
