# Supplementary material for: Pseudomonas fluorescens F113 Can Produce a Second Flagellar Apparatus, Which Is Important for Plant Root Colonization
Source: Front Microbiol. 2016 Sep 22;7:1471. doi: 10.3389/fmicb.2016.01471 (PMC5031763; doi:10.3389/fmicb.2016.01471)
Supplement: Supplementary file 3 [file Table_3.PDF]

Supplementary Table 3. Closest homologue of the proteins present in the flagelar island of *P. fluorescens* F113 based on blastp.

Query	Hit	Taxon	PercIdentities	E-Value
protein MotD	gi|502033318|ref|WP_012701325.1|	*Azotobacter vinelandii* CA6	45.6	2.13336e-61
protein MotC	gi|502033319|ref|WP_012701326.1|	*Azotobacter vinelandii* CA6	83.6	6.95918e-151
protein FliC2	gi|737429151|ref|WP_035409837.1|	*Chromohalobacter israelensis*	62.3	1.29752e-136
putative O-linked N-acetylglucosamine transferase	gi|163261056|emb|CAP43358.1|	*Bordetella petrii*	45.1	0.0
UDP-glucose dehydrogenase	gi|737609878|ref|WP_035580530.1|	*Halomonas* sp. TG39a	75.6	0.0
protein FliA2	gi|502033347|ref|WP_012701347.1|	*Azotobacter vinelandii* CA6	65.8	4.73172e-107
hypothetical protein PSF113_0744	gi|502033350|ref|WP_012701349.1|	*Azotobacter vinelandii* CA6	43.3	2.64419e-33
protein FlhF2	gi|502033352|ref|WP_012701350.1|	*Azotobacter vinelandii* CA6	49.5	0.0
protein FlhA2	gi|502033354|ref|WP_012701351.1|	*Azotobacter vinelandii* CA	76.8	0.0
protein FlhB	gi|737492053|ref|WP_035471736.1|	Gammaproteobacteria bacterium MFB021	58.4	2.95441e-157
protein MotB2	gi|502033006|ref|WP_012701015.1|	*Azotobacter vinelandii* CA6	63.1	6.43772e-129
protein MotA2	gi|502033005|ref|WP_012701014.1|	*Azotobacter vinelandii* CA6	77.0	1.51353e-152
protein FlhC	gi|502033004|ref|WP_012701013.1|	*Azotobacter vinelandii* CA6	80.7	1.01428e-99
protein FlhD	gi|502033003|ref|WP_012701012.1|	*Azotobacter vinelandii* CA6	62.5	1.82783e-23
protein FliT2	gi|502033000|ref|WP_012701009.1|	*Azotobacter vinelandii* CA6	44.0	6.84947e-17
protein FliS2	gi|502032999|ref|WP_012701008.1|	*Azotobacter vinelandii* CA6	60.6	3.04061e-33
protein FliD2	gi|502032998|ref|WP_012701007.1|	*Azotobacter vinelandii* CA6	51.8	4.51686e-126
protein FliE2	gi|512439444|ref|WP_016415264.1|	*Halomonas anticariensis* FP35 = DSM 16096	58.0	1.05151e-32
protein FliF2	gi|502032996|ref|WP_012701005.1|	*Azotobacter vinelandii* CA6	61.4	0.0
protein FliG2	gi|502032995|ref|WP_012701004.1|	*Azotobacter vinelandii* CA6	83.2	0.0
protein FliH2	gi|517848213|ref|WP_019018421.1|	*Halomonas lutea*	43.3	5.64843e-53
protein FliI2	gi|502032993|ref|WP_012701002.1|	*Azotobacter vinelandii* CA6	80.5	0.0
protein FliJ2	gi|502032992|ref|WP_012701001.1|	*Azotobacter vinelandii* CA6	42.8	3.63274e-29
protein FliK2	gi|502032991|ref|WP_012701000.1|	*Azotobacter vinelandii* CA6	32.6	5.39619e-29
protein FliL2	gi|648608688|ref|WP_026300439.1|	*Halomonas lutea*	41.0	6.65218e-32
protein FliM2	gi|502032989|ref|WP_012700998.1|	*Azotobacter vinelandii* CA6	76.9	0.0
protein FliN2	gi|517848206|ref|WP_019018414.1|	*Halomonas lutea*	68.5	4.52345e-63
protein FliO2	gi|502032987|ref|WP_012700996.1|	*Azotobacter vinelandii* CA6	49.6	7.59532e-28
protein FliP2	gi|737604898|ref|WP_035575553.1|	*Halomonas zincidurans*	78.4	1.00219e-113
protein FliQ2	gi|515482426|ref|WP_016915694.1|	*Halomonas stevensii*	64.8	2.91226e-33
protein FliR2	gi|517848202|ref|WP_019018410.1|	*Halomonas lutea*	61.5	5.60236e-108
protein FlgL2	gi|502032983|ref|WP_012700992.1|	*Azotobacter vinelandii* CA6	53.6	5.41077e-142
protein FlgK2	gi|502032982|ref|WP_012700991.1|	*Azotobacter vinelandii* CA6	57.0	0.0
protein FlgJ2	gi|502032981|ref|WP_012700990.1|	*Azotobacter vinelandii* CA6	55.2	7.20999e-101
protein FlgI2	gi|502032980|ref|WP_012700989.1|	*Azotobacter vinelandii* CA6	74.9	0.0
protein FlgH2	gi|502032979|ref|WP_012700988.1|	*Azotobacter vinelandii* CA6	72.1	3.11373e-100
protein FlgG2	gi|502032978|ref|WP_012700987.1|	*Azotobacter vinelandii* CA6	78.1	1.13238e-146
protein FlgF2	gi|502032977|ref|WP_012700986.1|	*Azotobacter vinelandii* CA6	67.7	1.4702e-112
protein FlgE2	gi|502032976|ref|WP_012700985.1|	*Azotobacter vinelandii* CA6	63.6	1.20514e-161
protein FlgD2	gi|512439466|ref|WP_016415286.1|	*Halomonas anticariensis* FP35 = DSM 16096	50.0	2.4484e-66
protein FlgC2	gi|512439467|ref|WP_016415287.1|	*Halomonas anticariensis* FP35 = DSM 16096	79.9	1.91031e-71
protein FlgB2	gi|502032973|ref|WP_012700982.1|	*Azotobacter vinelandii* CA6	73.0	9.3176e-63
protein FlgA2	gi|502032972|ref|WP_012700981.1|	*Azotobacter vinelandii* CA6	45.2	5.5106e-54
protein FlgM2	gi|502032971|ref|WP_012700980.1|	*Azotobacter vinelandii* CA6	50.9	1.09917e-07
protein FlgN2	gi|502032970|ref|WP_012700979.1|	*Azotobacter vinelandii* CA6	50.0	2.49522e-37
