# Supplementary material for: Pseudomonas fluorescens F113 Can Produce a Second Flagellar Apparatus, Which Is Important for Plant Root Colonization
Source: Front Microbiol. 2016 Sep 22;7:1471. doi: 10.3389/fmicb.2016.01471 (PMC5031763; doi:10.3389/fmicb.2016.01471)
Supplement: Supplementary file 4 [file Presentation_1.PDF]

Script 1. Blast parser

```
#! /usr/bin/python
from Bio.Blast import NCBIXML
import sys
import re

output = open("f113vsnr2xmlb.tab", 'w')
output.write('Query\tHit\tTaxon\tCoverage\tIdentities\tPercIdentities\tPositives\tP
ercPositives\tE-Value\tBit Score\n')
result_handle = open("f113vsnr2.xml")
blast_records = NCBIXML.parse(result_handle)
for blast_record in blast_records:
   for alignment in blast_record.alignments:
      queryprot = blast_record.query
      squery = re.match('(.+)( \[)', queryprot)
      query = squery.group(1)
      hitprot = alignment.hit_def
      searchobj = re.match('(gi\|.+?\|.+?\|.+?\|)(.+\[)(.+)(\])', hitprot)
      protein=searchobj.group(1)
      specie = searchobj.group(3)
      for hsp in alignment.hsps:
         identities = hsp.identities
         percidentities=round(float(identities)/float(hsp.align_length)*100,1)
         coverage = round(float(alignment.length) / float(hsp.align_length) * 100, 1)
         positives = hsp.positives
         percpositives=round(float(positives)/float(hsp.align_length)*100, 1)
         evalor= hsp.expect
         score= hsp.score
         output.write(str(query) + '\t' + str(protein) + '\t' + str(specie) + '\t' +
str(coverage) + '%\t' + str(identities) + '\t' +
                  str(percidentities) + '\t' + str(positives) + '\t' + str(percpositives) + '\t'+
str(evalor) + '\t' + str(score) '\n')
```

Script 2. Concatenation of sequences

```
#!/usr/bin/Python
import sys
import os
import re

r16S = open('r16S', 'r')
gyrB = open('gyrB', 'r')
rpoB = open('rpoB', 'r')
rpoD = open('rpoD', 'r')
out = open('output', 'w')

with open('del_16S', 'w') as del_16S:
	for line in r16S:
		line = line.rstrip('\n')
		if '>' in line:
			line = '\n'+line+'|'
			del_16S.write(line)
		else:
			del_16S.write(line)
del_16S.closed

with open('del_gyrB', 'w') as del_gyrB:
	for line in gyrB:
		line = line.rstrip('\n')
		if '>' in line:
			line = '\n'+line+'|'
			del_gyrB.write(line)
		else:
			del_gyrB.write(line)
del_gyrB.closed

with open('del_rpoB', 'w') as del_rpoB:
	for line in rpoB:
		line = line.rstrip('\n')
		if '>' in line:
			line = '\n'+line+'|'
			del_rpoB.write(line)
		else:
			del_rpoB.write(line)
del_rpoB.closed

with open('del_rpoD', 'w') as del_rpoD:
	for line in rpoD:
		line = line.rstrip('\n')
		if '>' in line:
```

```
				line = '\n'+line+'|'
				del_rpoD.write(line)
			else:
				del_rpoD.write(line)
del_rpoD.closed

del_16S_r = open('del_16S', 'r')
del_gyrB_r = open('del_gyrB', 'r')
del_rpoB_r = open('del_rpoB', 'r')
del_rpoD_r = open('del_rpoD', 'r')

for a, b, c, d in zip(sorted(del_16S_r), sorted(del_gyrB_r), sorted(del_rpoB_r),
sorted(del_rpoD_r)):
	if re.match('^$', a):
		pass
	else:
		match_16S = re.match('(>(.*))\|(.*)', a)
		match_gyrB = re.match('(>(.*))\|(.*)', b)
		match_rpoB = re.match('(>(.*))\|(.*)', c)
		match_rpoD = re.match('(>(.*))\|(.*)', d)

	out.write(match_16S.group(1)+'\n'+match_16S.group(3)+match_gyrB.group(3)
+match_rpoB.group(3)+match_rpoD.group(3)+'\n')
```
