# Supplementary material for: Pseudomonas fluorescens F113 Can Produce a Second Flagellar Apparatus, Which Is Important for Plant Root Colonization
Source: Front Microbiol. 2016 Sep 22;7:1471. doi: 10.3389/fmicb.2016.01471 (PMC5031763; doi:10.3389/fmicb.2016.01471)
Supplement: Supplementary file 6 [file Image_2.PDF]

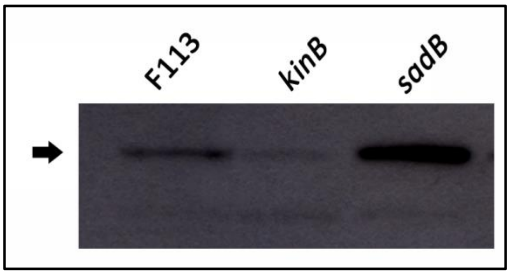

Supplementary Figure 2. Western blot analysis of extracelular proteins from *P. fluorescens* F113 and its *kinB* and *sadB* isogenic mutants. Blot was probed with an anti-FliC antiserum. The *sadB* mutant, but not the *kinB* mutant, showed overproduction of the flagellin FliC.
