# Supplementary material for: Pseudomonas fluorescens F113 Can Produce a Second Flagellar Apparatus, Which Is Important for Plant Root Colonization
Source: Front Microbiol. 2016 Sep 22;7:1471. doi: 10.3389/fmicb.2016.01471 (PMC5031763; doi:10.3389/fmicb.2016.01471)
Supplement: Supplementary file 5 [file Image_1.PDF]

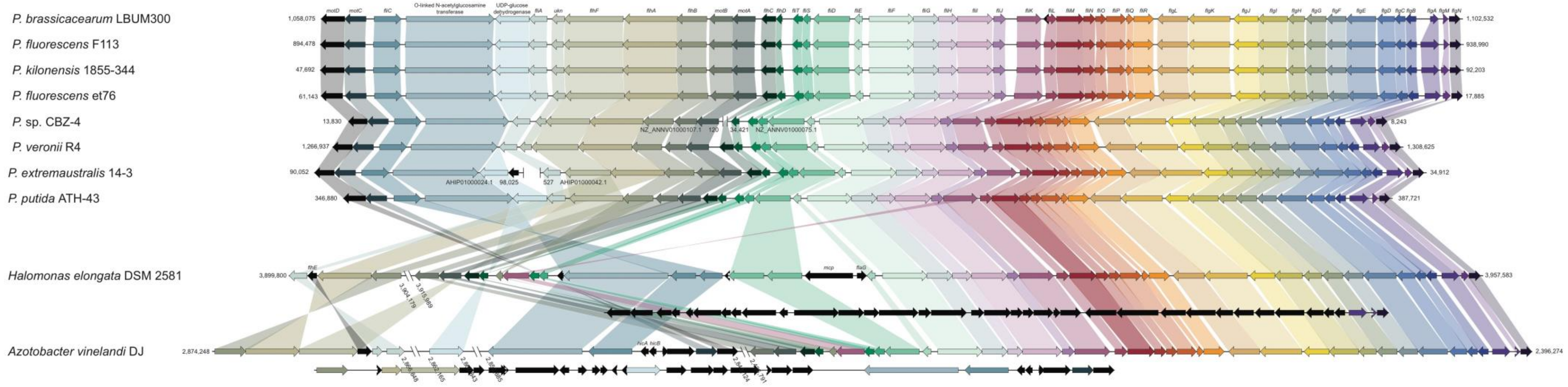

Supplementary Fig. 1. Genetic organization of the flagelar island in different *Pseudomonas* strains. The genetic organization is compared with the flagellar regions of *Azotobacter vinelandii* DJ and *Halomonas elongata* DSM2581.
